# Hiatal hernia

**DOI:** 10.11604/pamj.2016.24.40.9037

**Published:** 2016-05-10

**Authors:** Ebaa Samkari, Meshari Alshalawi

**Affiliations:** 1 Umm Al Qura University, Makkah, Saudi Arabia

**Keywords:** Hiatal hernia, bronchial asthma, chronic pancreatitis

## Image in medicine

61 years old known case of bronchial asthma, chronic pancreatitis, presented with postprandial epigastric campy abdominal pain and vomiting for one month no history of trauma. Initial evaluation revealed paraesophageal hernia. EGD finding is grade II esophagitis with modular mucosa and superficial ulceration, Distal part of the funds, body and the Antrim were rolled back into thoracic cavity. Abdominal computed tomography (CT) showed undulating diaphragm and large complex hiatal hernia. The cardia is above the diaphragm. The entire stomach is in the chest, paraesophageal, right and left to esophagus. The patient is symptomatic and surgery done for him.

**Figure 1:**
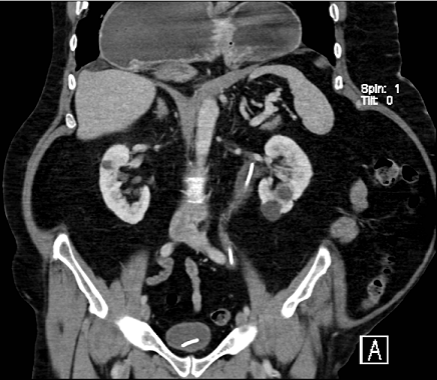
Abdominal computed tomography

